# Psychometric properties of the Attitudes and Beliefs about Sleepy Driving Scale in Chinese drivers and its relationships with driving behaviours

**DOI:** 10.1371/journal.pone.0269312

**Published:** 2022-06-02

**Authors:** Chunyue Zhao, Long Sun, Changlu Zhang

**Affiliations:** 1 School of Psychology, Liaoning Normal University, Dalian, Liaoning, P. R. China; 2 School of Education, Shenyang Normal University, Shenyang, Liaoning, P. R. China; Tsinghua University, CHINA

## Abstract

**Purpose:**

The present study aimed to adapt the Attitudes and Beliefs about Sleepy Driving Scale (ABSDS) to a sample of Chinese drivers and to examine its reliability and validity.

**Methods:**

Five hundred and twenty drivers aged 18 to 56 years old were asked to complete the ABSDS and a validated Chinese version of the Prosocial and Aggressive Driving Inventory.

**Results:**

The results showed that the final Chinese version of the ABSDS contained 7 items with satisfactory reliability. Second, significant gender differences were found in attitude towards sleepy driving, with female drivers scoring higher than male drivers. Third, significant correlations between ABSDS score and prosocial and aggressive driving behaviours were found. More importantly, ABSDS score can significantly predict drivers’ prosocial driving behaviours. Moreover, ABSDS score can significantly predict drivers’ violation involvement and accident involvement.

**Conclusion:**

The findings supported the psychometric properties of the Chinese version of the ABSDS and suggested that it can be used to assess drivers’ attitudes and beliefs about sleepy driving in China.

## Introduction

Sleepy driving is a major obstacle to creating a safe traffic environment. A systematic review and meta-analysis found that the probability of road traffic accidents for sleepy drivers is 1.29~1.34 times as high as that of non-sleepy drivers [1]. In China, sleepy driving or poor sleep quality as a threat to road safety has attracted the attention of many researchers [2, 3]. Sleepy driving behaviour is multifactorial in nature, and driver attitude is one of the important factors [4]. Many studies have demonstrated that a driver’s attitude towards risky driving behaviours (including sleepy driving) is closely associated with an increased frequency of risky behaviours while driving [5, 6]. For example, one study found that 81% of drivers believed that driving while sleepy has an adverse impact on their ability to drive safely, and 80% of drivers thought sleepy driving impaired their judgement while driving [7]. Therefore, measuring drivers’ attitudes towards sleepy driving is crucial for driving safety.

To date, many instruments using self-reported methods have been developed to measure attitudes and beliefs towards sleepy driving [8, 9]. For example, in one study, a series of closed-ended questions was designed to probe respondents’ attitudes, concerns, and self-reported behaviours related to sleepy driving [8]. These variables were also measured by asking subjects to complete five posture items (comprised of five pairs of bipolar adjectives) under three drowsy driving situations [9]. Moreover, the Attitudes and Beliefs about Sleepy Driving Scale (ABSDS) and driving diaries were used to measure driving attitudes [7]. The ABSDS revealed that the majority of participants believed that individuals should not drive while falling asleep and understand the consequences of sleepy driving, but they thought that sleepiness had little effect on their decision-making and distractibility. This study aimed to adapt the ABSDS to the Chinese language and examine its reliability and validity to provide a valid instrument for assessing drivers’ attitudes towards sleepy driving in China.

### Attitude towards sleepy driving, driving behaviours and traffic violations

Previous studies have found that sleepiness or drowsiness during driving is an important factor leading to dangerous driving behaviours and traffic accidents [1, 4, 10]. Studies have found that sleepy driving or feeling drowsy while driving is associated with slower detection of road hazards [11, 12], frequent distracted driving [13] and impairment of vehicle control [14]. Sleepiness-related accidents often occur as a consequence of driving errors. Sleepy drivers cannot compensate, resulting in effects such as deterioration of steering quality, a reduction in lane-keeping accuracy, and drivers becoming out of the control loop during automated driving [15].

However, only a few studies have examined the relationships between attitudes towards sleepy driving and risky driving behaviours and traffic accidents. Studies found that drivers who reported more favourable attitudes towards sleepy driving behaviour were more likely to engage in sleepy driving behaviours [16, 17]. A second study found that participants thought sleepy driving behaviour was less dangerous than driving with a high blood alcohol concentration, and drivers who believed that sleepy driving behaviour was less dangerous were more likely to perform the behaviour [4]. However, other studies have found that attitude about sleepy driving cannot predict traffic violations, as people’s worries intensify when they think that sleepy driving is dangerous; as a result, drivers’ sleepy driving behaviour may decrease [8]. For example, one study found that drivers showed more avoidance behaviours under sleepy conditions than under sober conditions [7]. Given the inconsistency in the literature, the relationship between attitudes towards sleepy driving and traffic accidents needs to be further investigated.

Despite the associations between drivers’ attitudes towards sleepy driving and risky driving behaviours [16, 17], few studies have explored safe or prosocial driving behaviours and their associations with attitudes towards road safety. Prosocial driving behaviour is a safe driving behaviour that protects the well-being of road users [18]. To better establish the relationship between drivers’ attitudes towards sleepy driving and safe and unsafe driving behaviours, the Prosocial and Aggressive Driving Inventory (PADI) was used in this study to validate the ABSDS.

### Attitude towards sleepy driving and demographic factors

Regarding the associations between demographic factors and attitude towards sleepy driving, mixed results have been reported. Gender differences in attitudes towards sleepy driving were found, with male drivers showing a more favourable attitude towards sleepy driving than female drivers [19]. One study found that the more objective the attitude towards sleepy driving among female drivers was, the more prosocial driving behaviour they have [20]. Regarding age, compared to older drivers, younger drivers reported more positive attitudes towards sleepy and drunk driving behaviours, and younger drivers were also more likely to report exhibiting sleepy driving behaviours than older drivers [16, 21]. However, other studies found that age is negatively related to drivers’ attitudes towards sleepy driving [22]. Older age and the belief of having a perceived ability to overcome sleepiness results in an increased likelihood of continuing to drive while sleepy [22]. Thus, the present study aimed to explore the relationships between demographic factors and sleepy driving attitudes in a sample of Chinese drivers.

### Aims of the present study

This study aimed to adapt and validate the ABSDS in Chinese drivers and assess its reliability by examining its relationships with traffic violations, aggressive and prosocial driving behaviours. It was assumed that the higher a driver’s ABSDS score is, the more likely they are to exhibit prosocial driving behaviour and less likely they are to exhibit aggressive driving behaviour and that ABSDS score is negatively related to traffic violations and accidents.

## Methods

### Participants and procedures

The present study was approved by the Logistics Department for Civilian Ethics Committee of Liaoning Normal University. Participants were recruited in supermarket such as Warmart and Carrefour in Dalian, Shenyang, Shanghai and Chengdu. The inclusion criteria were having a valid driving license and being at least 18 years old. Data were collected from November 6 to 28 in 2020. Upon reading the ads, shoppers who were interested in this study took our paper-pencil survey. Participants were first told the purpose of the study and signed a consent form. Then, the participants completed ABSDS, PADI and a demographic questionnaire within 30 minutes. When the participants finished the questionnaires, each received a gift coupon (5 RMB) as compensation.

Five hundred and twenty private car drivers with driving licences took part in this study. Data from 15 participants were determined to be invalid due to the absence of an answer and were excluded. The final sample consisted of 237 male drivers (46.9%) and 268 female drivers (53.1%). Participants ranged in their age from 18 to 56 years old (*M* = 32.05, *SD* = 11.17). All participants had possessed a valid driving licence for more than 0.5 years (*M* = 4.94, *SD* = 6.18). Number of feeling sleepy while driving per week ranged from 0 to 7 times (*M* = 0.48, *SD* = 1.13). A total of 16.6% of the participants were involved in at least one traffic violation, and the other 83.4% were not involved in any traffic violation while driving in the previous 12 months. Fifteen percent of participants reported having traffic accidents, and 85% did not have accidents while driving in the previous 12 months. Detailed demographic information of the participants is shown in Table 1.

**Table 1:** Demographic information of participants.

Demographic information	Min	Max	Mean	SD
Age (yr)	18	56	32.05	11.17
Gender ^a^	1	2	1.53	0.50
Years of experience (yr)	0.50	30	4.94	6.18
Average driving time per week	0.25	8	1.31	1.15
Number of violations last 12 months	0	3	0.23	0.58
Number of accidents last 12 months	0	3	0.19	0.48
Number of feeling sleepy while driving per week	0	7	0.48	1.13

Note

^a^ 1 = male, 2 = female.

## Measures

### Attitudes and Beliefs about Sleepy Driving Scale

With the permission of the rights holder, the ABSDS was translated into Chinese. The original scale includes 7 items with satisfactory reliability [7]. The items assessed drivers’ attitudes towards sleepy driving behaviours and compared their attitudes towards sleepy driving and speeding behaviours while driving. Participants were asked to rate the degree to which they agreed each of the items on a 5-point Likert scale ranging from 1 (strongly disagree) to 5 (strongly agree).

According to the translation/back-translation procedure, one translator with a master’s degree in English translated the original scale into Chinese. Then, other translators translated the new version into English. The researchers and two translators compared the newly translated version with the original scale and then chose the most appropriate expression for each item. After choosing the appropriate translation, an initial Chinese version of the scale was obtained.

### Prosocial and Aggressive Driving Inventory (PADI)

The 28-item PADI is a self-report questionnaire with 2 factors [18]. Prosocial driving behaviours (16 items) are considered safe driving behaviours that could help protect the participants and other drivers from harm on roadways. Aggressive driving behaviours (12 items) are considered unsafe driving behaviours that could harm people who share on the road. The participants were asked to rate how often they exhibit each of these driving behaviours on a 6-point scale ranging from 1 (never) to 6 (always). In this study, the reliability was 0.93 for prosocial driving behaviours and 0.81 for aggressive driving behaviour.

### Demographic questionnaire

Participants also reported their demographic information on a self-report questionnaire, including gender, age, years since licensure, number of hours driven per week and number of feeling sleepy while driving per week. Participants also reported the number of traffic violations and accidents they had while driving in the previous 12 months.

### Statistical analysis

The data were analysed by SPSS version 18.0. First, exploratory factor analysis (EFA) was conducted to explore the factorial structure of the ABSDS. Second, Pearson correlations among ABSDS score, PADI factors, and the numbers of violations and accidents were analysed to explore the construct validity of the ABSDS. Third, gender differences in ABSDS scores were analysed using independent samples t-tests, and the relationships among ABSDS score and age, average driving time, and driving experience were examined using Pearson correlations. Third, hierarchical regression analyses were conducted to explore the ability of ABSDS score to predict prosocial and aggressive driving behaviours. Finally, the predictive role of the ABSDS score in whether a driver involved in traffic violations and accidents were examined using binary logistic regression analysis.

## Results

### Factor analysis

An EFA using primary axis extraction and varimax rotation was conducted. The results revealed a one-factor structure that can explain 74.82% of the variance (KMO = 0.92, Bartlett = 3201.16, *p* < 0.01). The item-total correlations, factor loading and Cronbach’s alpha if item deleted are reported in Table 2.

**Table 2:** The factor loadings and item test.

Items	Mean (SD)	Factor loading	Item test	Cronbach’s α if item deleted
5. My judgement is impaired if I drive while sleepy	4.02 (1.27)	0.91	0.91**	0.93
7. I tend to get easily distracted if I drive while sleepy	4.07 (1.26)	0.91	0.90**	0.93
6. Sleepy driving is more dangerous than speeding	4.10 (1.27)	0.89	0.89**	0.93
4. Driving under the influence of alcohol or drugs is more dangerous than sleepy driving	4.10 (1.32)	0.87	0.86**	0.93
2. A driver should not drive if he or she is sleepy	4.20 (1.28)	0.86	0.86**	0.93
1. Driving while sleepy impacts on my ability to drive safely	3.80 (1.42)	0.81	0.82**	0.94
3. I make bad driving decisions if I drive while sleepy	3.74 (1.42)	0.80	0.81**	0.94

Note

*p < 0.05

**p < 0.01.

### Reliability of the ABSDS

Reliability analyses showed that the Cronbach’s alpha of the scale was 0.94, indicating that the reliability of the ABSDS was acceptable.

### Effects of demographic factors on ABSDS score

Regarding gender differences in ABSDS scores, independent samples t-tests (see Table 3) showed that male drivers’ scores related to attitudes towards sleepy driving were significantly lower than female drivers. Specifically, female drivers were more likely to agree that sleepy driving has an adverse effect on their driving ability and leads to distracted driving and risky driving behaviours.

**Table 3:** Mean and deviation of the ABSDS items by gender.

Items	Male (*n* = 237)		Female (*n* = 268)		*t*	*Cohen’s d*
	M	SD	M	SD		
1	3.65	1.43	3.94	1.41	-2.33*	-0.21
2	4.00	1.36	4.38	1.18	-3.29**	-0.29
3	3.66	1.42	3.81	1.43	-1.17	-0.10
4	3.99	1.36	4.21	1.28	-1.86	-0.17
5	3.91	1.30	4.11	1.24	-1.81	-0.16
6	3.92	1.34	4.25	1.18	-2.94**	-0.26
7	3.95	1.27	4.18	1.24	-2.02*	-0.18
Total score	3.87	1.19	4.12	1.08	-2.54*	-0.23

Note

*p < 0.05

**p < 0.01.

Pearson correlations showed that the participants’ age and years of driving experience were positively correlated with the total score (*r* = 0.17, *p* < 0.01; *r* = 0.13, *p* < 0.01). No significant correlations were found between the total score and weekly driving time or the number of feeling sleepy while driving per week.

### Validity of the ABSDS

To examine the validity of the Chinese version of the ABSDS, the correlations between ABSDS score and PADI factors, the number of self-reported traffic violations and the number of accidents in the previous 12 months were analysed. The results are shown in Table 4.

**Table 4:** Correlations between the ABSDS, PADI factors and traffic violations.

Variables	1	2	3	4	5
ABSDS score (1)	-				
Aggressive driving behaviours (2)	-0.30**	-			
Prosocial driving behaviours (3)	0.54**	-0.32**	-		
Number of violations (4)	-0.16**	0.12**	-0.08	-	
Number of accidents (5)	-0.12***	0.09**	-0.06	0.93**	-

Note

*p < 0.05

**p < 0.01.

Table 4 shows that the ABSDS score was significantly and positively correlated with prosocial driving behaviour and negatively correlated with aggressive behaviours, the number of violations and the number of accidents. The results showed that the discriminant validity of the ABSDS was acceptable.

### Ability of the ABSDS score to predict driving behaviours

To explore whether ABSDS score can predict driving behaviours, hierarchical regression analyses (enter method) were conducted with prosocial and aggressive driving behaviours as dependent variables. In the first step, demographic variables (gender, age, years of driving experience, and average driving time) were entered; ABSDS score was entered in step two. The results are shown in Table 5.

**Table 5:** Hierarchical regression coefficients for prediction of driving behaviours.

Variable	Prosocial driving behaviours		Aggressive driving behaviours	
	*β*	*t*	*β*	*t*
**Step one**				
Gender	0.15	3.92**	-0.18	-4.71**
Age	0.13	2.33*	-0.06	-0.97**
Experience	0.08	1.47	-0.03	-0.45
Weekly driving time	-0.03	-0.60	-0.07	-1.44
*Δ R* ^*2*^	0.097**		0.057**	
**Step two**				
ABSDS score	0.49	13.21**	-0.26	-5.98*
*Δ R* ^*2*^	0.233**		0.061**	
Total *Δ R*^*2*^	0.330**		0.118**	

Note

*p < 0.05

**p < 0.01.

Table 5 shows that ABSDS score can significantly predict prosocial driving behaviours and aggressive driving behaviours, explaining 23.3% and 6.1% of the variance, respectively.

### ABSDS score and traffic violations and accidents

The predictive role of the ABSDS score in whether a driver involved in traffic violations or traffic accidents were examined using binary logistic regression analysis. Demographic variables entered as controlled variables and the ABSDS score entered as independent variables. The results (see Table 6) show that ABSDS score is effective predictor of violation involvement (OR = 1.499, 95% CI = 1.198–1.875) and accident involvement (OR = 1.561, 95% CI = 1.236–1.972).

**Table 6:** Predictive role of ABSDS score on accident involvement.

Variables	Violation involvement			Accident involvement		
	Odds ratio	95% CI	*p*	Odds ratio	95% CI	*p*
Gender	1.653	0.966–2.832	0.067	1.601	0.911–2.814	0.102
Age	0.973	0.901–0.965	0.001	0.928	0.895–0.962	0.001
Experience	0.967	0.922–1.014	0.168	0.966	0.920–1.014	0.167
Weekly driving time	0.777	0.628–0.961	0.020	0.771	0.620–0.959	0.020
ABSDS score	1.499	1.198–1.875	0.001	1.561	1.236–1.972	0.001

## Discussion

In this study, the Attitudes and Beliefs about Sleepy Driving Scale was translated and adapted to a Chinese driver sample and the results showed its reliability and validity were acceptable.

First, the psychometric properties of Chinese version of the ABSDS were satisfactory. The results obtained for the Chinese version of the ABSDS were similar to the results obtained in the Australian sample [7]. All items yielded high loadings on a single dimension. The reliability of the whole scale was 0.94. The mean and standard deviations of each item and the reliability of the scale were similar to those reported in the original study [7]. These results confirmed that the Chinese ABSDS has adequate psychometric properties and can be used as a useful tool for evaluating attitudes towards sleepy driving.

Second, the results of this study show that male are more tolerant and have a more favourable attitude towards sleepy driving than female. A growing body of evidence shows that being a male driver consistently increases the chance of sleeping behind the wheel [23]. However, no gender differences were found from item 3 to item 5. These results were supported by the findings of previous studies showing that alcohol impairs drivers’ perceptions more than feeling sleepy [24], and the dangers of driving drowsily are more likely to be downplayed or ignored than those of drunk or drug driving [13]. This study found that younger, less experienced male drivers were more tolerant of sleepy driving. That is, increases in age and driving experience make drivers opposed to sleepy driving. Previous studies have found that younger drivers have a more positive attitude towards sleepiness and drunk driving compared to older drivers [21], and young drivers’ supportive attitudes towards sleepy driving are effective predictor of sleepy-related behaviours and traffic accidents [25]. There are contradictory results showing that drivers’ age is positively correlated with sleepy driving behaviours in a sample of 137 young drivers aged 18 to 25 years [16, 22]. One possible explanation is that younger drivers (18–25 years old) have a limited amount of driving experience, including driving while sleepy and driving at night, which could result in newly licensed drivers deciding not to engage in sleepy driving behaviours.

Furthermore, ABSDS score is positively correlated with prosocial driving behaviours and negatively correlated with aggressive driving behaviours, showing its discriminant validity was acceptable. Notably, the scale score can positively predict prosocial driving behaviours and negatively predict aggressive driving behaviours. Studies have shown that drivers’ age and attitude have more influence on drowsy driving than personality traits or risk-taking factors, and a positive attitude towards drowsy driving makes drivers choose prosocial driving behaviours such as parking to ensure the safety of other drivers [26, 27]. Although many studies have demonstrated that sleepy driving leads to more risky driving and accidents, this study found that drivers who support sleepy driving exhibit fewer prosocial behaviours. The findings provide a new perspective for enhancing driving safety in China by taking attitudes towards sleepy driving into consideration when interventions were developed.

Finally, this study found that drivers’ approval of sleepy driving is positively correlated with their numbers of traffic violations and accidents, although few participants reported traffic violations (16.6%) or traffic accidents (15%) in the past year. More importantly, ABSDS score can significantly predict drivers’ violation and accident involvement in last 12 months. One possible explanation is that a lower perceived legitimacy and ignoring the dangers of sleepy driving or having a perceived ability to overcome sleepiness lead to more violations and accidents [5, 28]. The results show direct associations between drivers’ attitudes towards sleepy driving and the number of traffic accidents and can be used to develop interventions targeted at attitudes towards sleepy driving in China.

Given driver education initiatives with respect to sleepy driving are limited in China; one strategy is to make the testing of knowledge about sleepy driving a compulsory part in driver education and assessment. For instance, drivers whose driving licenses were suspended due to traffic violations and accidents that related to sleepy driving (fines up to 12 points) must attend safety education to re-obtain their licenses. During this session, measures of those drivers’ attitudes toward sleepy driving and the changes of their attitudes that sleepy driving is acceptable might help improve the effectiveness of driver education. Studies have found that an effective behaviour change approach requires the discovery of a determinant of predicted behaviour, the ability to change that determinant, and the translation into practical application in a way that is appropriate to the target population [29]. Given the results obtained in the present study, attitude about sleepy driving can be a determinant that could be easily evaluated and monitored in different phrases of driver education. Another promising way is to teach drivers who are prone to traffic violations and accidents to identify the signs of sleepiness while driving and to learn some sleepiness countermeasures, as evidence have shown that moderately sleep deprived individuals can identify increasing sleepiness and then take action to cease a hazard perception task [12, 30].

### Limitations and implications

The current study has some limitations. First, the data in the present study were collected via self-reported methods. However, as in previous studies [7–9], self-reported methods seemed to be the most suitable when exploring drivers’ attitude, motivation and driving behaviours. Future studies should include more objective measure of sleepy-related driving behaviours. Second, although the present study found that drivers’ attitudes and beliefs about sleepy driving are related to the number of traffic accidents, a follow-up study is needed to reveal the influences of attitudes and beliefs about sleepy driving on crash risk in the future. For instance, researchers can further explore the predictive validity of the ABSDS on drivers’ crash risk in a six-month or one-year period. Finally, the present study recruited 520 private car drivers of varying driving experience and age from four cites that geographically represent Chinese cities, which is enough given the main purpose of this study is to revise the 7-item ABSDS. However, future studies with larger samples are needed to further provide more evidence regarding the validity of the newly-developed scale for Chinese drivers.

The findings of the present study have some implications. Firstly, this study provides a reliable and validate instrument for assessing Chinese drivers’ attitudes toward sleepy driving. Although objective data about the attitudes toward sleepy driving is valuable, the consistency between objectively and subjectively measured attitudes toward sleepy driving might provide a new perspective in driver assessment and education. In this regard, the 7-item scale could serve as a short and time-saving tool when it was incorporated into the assessment of attitudes toward sleepy driving. Secondly, the newly-developed scale can be used to identify drivers who are prone to traffic accidents, thus helping strengthening injury prevention in China. Consistent with the findings of previous studies [4, 16], the present study found that drivers who showed more supportive attitude towards sleepy driving have a higher chance to be involved in traffic violations and accidents than those drivers who thought sleepy driving is dangerous.

## Conclusions

In conclusion, the Chinese version of the ABSDS has acceptable reliability and validity, providing a short and highly useful measurement of attitudes towards sleepy driving in China. Our findings show that drivers’ attitudes towards sleepy driving are associated with prosocial and aggressive driving behaviours. Drivers with high scores exhibit more prosocial driving behaviours and less aggressive driving behaviours. The associations between ABSDS score and traffic accidents also highlight that safety education and interventions targeting at changing drivers’ supportive attitudes towards sleepy driving are beneficial for enhancing road safety in China.
